# *MSH6* and *PMS2* germ-line pathogenic variants implicated in Lynch syndrome are associated with breast cancer

**DOI:** 10.1038/gim.2017.254

**Published:** 2018-01-18

**Authors:** Maegan E Roberts, Sarah A Jackson, Lisa R Susswein, Nur Zeinomar, Xinran Ma, Megan L Marshall, Amy R Stettner, Becky Milewski, Zhixiong Xu, Benjamin D Solomon, Mary Beth Terry, Kathleen S Hruska, Rachel T Klein, Wendy K Chung

**Affiliations:** 1 GeneDx, Gaithersburg, USA Maryland; 2 Department of Epidemiology, Columbia University, New York, New York USA; 3 Departments of Pediatrics and Medicine, Columbia University, New York, New York USA

**Keywords:** breast cancer, Lynch syndrome, mismatch repair, *MSH6*, *PMS2*

## Abstract

**Purpose:**

An association of Lynch syndrome (LS) with breast cancer has been long suspected; however, there have been insufficient data to address this question for each of the LS genes individually.

**Methods:**

We conducted a retrospective review of personal and family history in 423 women with pathogenic or likely pathogenic germ-line variants in *MLH1* (*N* = 65), *MSH2* (*N* = 94), *MSH6* (*N* = 140), or *PMS2* (*N* = 124) identified via clinical multigene hereditary cancer testing. Standard incidence ratios (SIRs) of breast cancer were calculated by comparing breast cancer frequencies in our study population with those in the general population (Surveillance, Epidemiology, and End Results 18 data).

**Results:**

When evaluating by gene, the age-standardized breast cancer risks for *MSH6* (SIR = 2.11; 95% confidence interval (CI), 1.56–2.86) and *PMS2* (SIR = 2.92; 95% CI, 2.17–3.92) were associated with a statistically significant risk for breast cancer whereas no association was observed for *MLH1* (SIR = 0.87; 95% CI, 0.42–1.83) or *MSH2* (SIR = 1.22; 95% CI, 0.72–2.06).

**Conclusion:**

Our data demonstrate that two LS genes, *MSH6* and *PMS2*, are associated with an increased risk for breast cancer and should be considered when ordering genetic testing for individuals who have a personal and/or family history of breast cancer.

## Introduction

Germ-line pathogenic variants (PVs) in the mismatch repair (MMR) genes *MLH1*, *MSH2*, *MSH6*, and *PMS2*, and 3′ deletions of *EPCAM*, a gene just upstream of *MSH2*, cause Lynch syndrome (LS). There is evidence that cancer risk depends on the affected gene. *MLH1* and *MSH2* are most commonly associated with a higher risk of colorectal cancer.^1^ Additionally, *MSH2* has been associated with a higher risk for extracolonic cancers, and female *MSH6* carriers may have the highest risk of endometrial cancer. While the LS tumor spectrum has been well established as including colon, endometrial, ovarian, stomach, small bowel, hepatobiliary tract, ureter/renal pelvis, pancreas, brain, and sebaceous neoplasms, the risks of other cancers, including breast, prostate, and adrenocortical tumors, are less clearly delineated.^1,2^

The MMR pathway is responsible for repairing single base pair mismatches and small insertions or deletions that occur when DNA polymerase attempts to replicate small repeat sequences. Pathogenic variants within the MMR genes result in errors in DNA repair, leading to an increased mutation load in MMR-deficient cells.^1^ Screening for MMR deficiency by microsatellite instability and immunohistochemistry assays is often performed on colorectal and endometrial tumors to identify patients with LS. Although screening for MMR deficiency in breast tumors is currently not standard clinical practice, multiple studies have shown that breast cancers from women with LS are more likely to exhibit microsatellite instability and loss of one or more MMR proteins via immunohistochemistry, compared with sporadic breast cancers.^3,4,5,6,7,8,9,10,11,12,13,14^

Clinical studies evaluating breast cancer risk in women with LS have been conflicting, with some showing up to a fourfold increased breast cancer risk and others reporting no increased risk.^15,16^ Ascertainment bias may have affected results: many previous LS studies assessing breast cancer ascertained their study cohorts based on criteria heavily weighted toward colorectal cancer. As a result, many study cohorts consisted only of *MLH1* and *MSH2* carriers although some also included *MSH6*.^17,18,19,20,21,22,23^ A few studies have included all four MMR genes, but presented only a single combined breast cancer risk, likely due to a small number of *MSH6* and *PMS2* carriers.^15,16^ Thus, the lack of consistent association of breast cancer and LS in previous studies may reflect the genetic composition of the cohorts. While it has been suggested that breast cancer risk may vary by gene in LS, no one study has examined gene- and age-specific breast cancer risks for all four MMR genes. The aim of our study was to characterize breast cancer risks for each MMR gene in our group of women who were identified to have a PV via germ-line hereditary cancer panel testing for a variety of cancer-related indications.

## Materials and methods

### Participants

We retrospectively queried more than 50,000 women who had multigene hereditary cancer panel testing completed at GeneDx between 2013 and 2016, identifying 423 women with a single germ-line pathogenic variant or likely pathogenic variant (collectively referred to here as PV) in any of the four MMR genes. All sequence variants were classified based on the 2015 American College of Medical Genetics and Genomics/Association for Molecular Pathology guidelines for the interpretation of sequence variants.^24^ Combined *EPCAM-MSH2* deletions were included in the *MSH2* PV cohort. Individuals with a second PV in an MMR or another gene (excluding single PV in *MUTYH*) were excluded. A list of the multigene panels (with gene lists) included and PVs identified can be found in Supplementary Tables S1 and S2 online.

Demographic, clinical, and family history information was obtained from test requisition forms and accompanying clinical documents supplied by the ordering provider at the time of the test order. Pathology reports were not requested to confirm diagnoses. Subjects were limited to females over the age of 18. Breast cancer was defined as any invasive breast neoplasm or ductal carcinoma *in situ*. This study was approved by the Western Institutional Review Board, Puyallup, WA (WIRB 20162523).

### Technical/laboratory methods

Genomic DNA was isolated from whole blood using a QIAsymphony DNA kit, and from oral rinse using a QIAsymphony DSP Virus/Pathogen Midi Kit (Qiagen, Valencia, CA). Genomic DNA was enriched for the complete coding region and splice-site junctions of the genes of interest using custom SureSelect targeted capture (Agilent, Santa Clara, CA). Next-generation sequencing and deletion/duplication analysis were performed for all coding regions as well as a portion of the 5′ untranslated region, 3′ untranslated region, and intronic regions for all genes on each panel, with the exception of EPCAM, for which only deletion/duplication analysis was performed. The products were sequenced on Illumina MiSeq or HiSeq instruments with paired-end reads (Illumina, San Diego, CA). DNA sequence was mapped to a masked version of the published human genome build University of California–Santa Cruz hg19/GRCh37 reference sequence using BWA-Mem version 0.7.8.^25,26^ Local realignment around insertion/deletion sites and regions with poor mapping quality was performed using the Genome Analysis Toolkit version 1.6 IndelRealigner.^27^ Variant calls were generated by the union of SAMtools version 0.1.18,^28^ Genome Analysis Toolkit UnifiedGenotyper,^27^ and a GeneDx-developed heuristic caller. Capillary sequencing (Applied Biosystems/Life Technologies, Grand Island, NY) on a newly extracted DNA sample was used to confirm all variants with clinical or uncertain significance and to fill in sequence for regions with fewer than 15 reads by next-generation sequencing. Any potential variant position with coverage of fewer than 50 reads was reviewed by analysts and analyzed by capillary sequencing if unclear. Long-range polymerase chain reaction was used to distinguish variants in *PMS2* from those in the *PMS2* pseudogene *PMS2CL*. Deletion/duplication analysis was performed via custom-designed exon-targeted array comparative genomic hybridization (Agilent). Confirmation of copy-number changes detected on array comparative genomic hybridization was performed by multiple ligation-dependent probe amplification, repeat microarray analysis, or quantitative polymerase chain reaction using the Universal ProbeLibrary (Roche, Indianapolis, IN).

### Statistical analysis

Standardized incidence ratios (SIRs) for breast cancer were calculated for each gene as the number of observed cancers to the number of expected cancers based on breast cancer incidence in the general population. The expected number of cases was estimated for each 5-year age group from 20 to 85 as the time at risk multiplied by the reference population incidence rates. The population-based incidence rates were obtained using SEER*Stat statistical software from the Surveillance, Epidemiology, and End Results (SEER) 18 data, which includes 18 cancer registries representing 27.8% of the US population.^29,30^ Corresponding 95% confidence intervals (95% CI) were calculated using the Poisson approximation distribution. SIRs for endometrial, colorectal, and ovarian cancer were also calculated using their respective incidence rates from SEER 18. All statistical analyses were conducted using RStudio 0.99.902.^31^ The Kaplan–Meier method was used to estimate cumulative risk and corresponding 95% CIs for breast cancers to age 60, stratified by MMR gene.^32^ Log-rank test was used to compare incidence rates between groups. All reported *p* values are two-tailed with a significance level of 0.05.

## Results

### Cohort characteristics

Most of the 423 female MMR PV carriers in our study reported Caucasian or European ancestry (72.1%, 305/423). The average age at time of genetic testing was 52.5 (±12.1) years (see Table 1 for further demographic details). The distribution of MMR PVs was as follows: *MSH6*, 33.1% (140/423); *PMS2*, 29.3% (124/423); *MSH2*, 22.2% (94/423); *MLH1*, 15.4% (65/423). The 423 women in our study harbored 241 unique PVs, the majority of which (87.5%, 370/423) were predicted to result in a truncated protein or a transcript subject to nonsense-mediated decay (start codon, splice, cryptic splice, nonsense, deletion/duplication, frameshift), while the rest were missense or small in-frame deletions (12.5%, 53/423) (Supplementary Figure S1).

**Table 1:** Demographic and clinical characteristics of study cohort

**Demographic/Clinical Characteristic**	**MMR PV Positive** *N* ** = 423**
**MMR gene, No. (%)**	
*MLH1*	65 (15.4)
*MSH2*	94 (22.2)
*MSH6*	140 (33.1)
*PMS2*	124 (29.3)
**Personal History**	
Personal Hx of Breast Cancer, No. (%)	107 (25.3)
Average Age at Breast Cancer Dx, year (SD)	50.2 (11.7)
Range of Ages at Breast Cancer Dx, years	26–76
Average Age at Testing, years (SD)	52.5 (12.1)
**Self-reported Race/Ethnicity, No. (%)** ^#^	
Caucasian/European	325 (83.8)
Hispanic	28 (7.2)
Black/African American	24 (6.2)
Asian/Pacific Islander	21 (5.4)
Native American	8 (2.1)
Other	5 (1.3)

No.=number; SD=standard deviation.

^#^Percentages represent proportion of women who provided race/ethnicity information (*N* = 388). Women reporting more than one race/ethnicity are counted more than once.

### Cancer history and referral patterns

A personal history of breast cancer was reported in 25.3% (107/423) of the cohort, with 1.4% (6/423) reporting a history of more than one primary breast cancer. The average age at first breast cancer diagnosis was 50.2 years (±11.7, range: 26–76).

Approximately half of the women with a MMR PV were referred for testing with either the Comprehensive Cancer Panel (24.6%, 104/423) or the Breast/Ovarian Cancer Panel (24.3%, 103/423), both of which contain genes related to breast cancer and LS (Supplementary Table S1). Twenty-six percent (27/104) of Comprehensive Cancer and 45.6% (47/103) of Breast/Ovarian Cancer Panel patients reported a personal history of breast cancer. Twenty-eight percent (117/423) of the women with MMR PVs were referred for the Colorectal Cancer Panel or the Lynch Syndrome Panel. Of these 117 women, 2 (1.7%) had breast cancer. Reflex testing (more extensive genetic testing after the first panel yielded normal results) identified PVs in three women with breast cancer following a normal result on the High-Risk Breast Cancer Panel.

**Figure 1:**
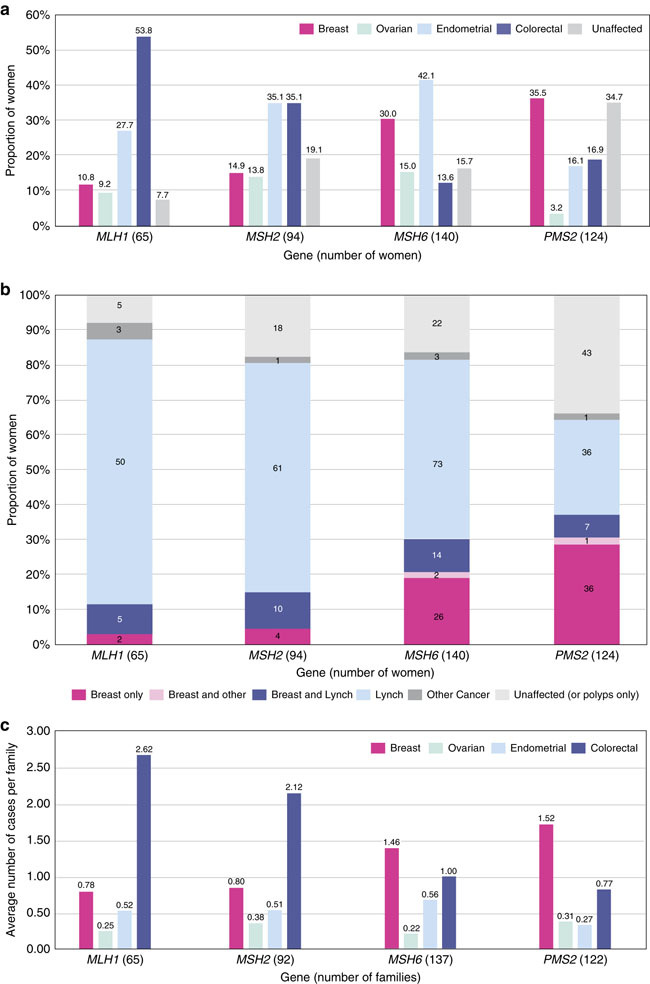
Personal and family history. (**a**) Personal history of breast and Lynch syndrome (LS)-associated cancers. Women with multiple cancers are counted more than once. Fallopian tube and primary peritoneal cancer were both included as ovarian cancer. Unaffected: no history of cancer but could have a history of colorectal polyps. (**b**) Personal history: breast cancer only versus other histories. Individuals are counted only once. Breast only: breast cancer, but no history of a LS-associated or any other cancer; Breast and Lynch: breast cancer plus a LS-associated tumor; Breast and other: breast cancer and possibly another non-LS-associated tumor; Lynch: LS-associated tumor and possibly other cancers; Other cancer: history of cancer that is not breast or a LS-associated tumor. (**c**) Family history: average number of cancer cases per family. Includes family history data only, not probands. Seven individuals were related to someone else in our cohort, but their families were only counted once. Total families = 416.

Prevalence of breast, ovarian, endometrial, and colorectal cancer varied among *MLH1*, *MSH2*, *MSH6*, and *PMS2* carriers (Figure 1a). After preliminary analyses, genes were grouped based on similar breast cancer prevalence observed in MMR PV carriers (*MLH1/MSH2* vs. *MSH6/PMS2*). A diagnosis of breast cancer, irrespective of other personal cancer history, was reported more frequently in women with *MSH6* or *PMS2* PVs (*MSH6*: 30%, 42/140; *PMS2*: 35.5%, 44/124) compared to women with *MLH1* or *MSH2* PVs (*MLH1*: 10.8%, 7/65; *MSH2*: 14.9%, 14/94) (*p* < 0.001) (Figure 1a). Furthermore, breast cancer in the absence of LS-associated cancers was reported more frequently in women with PVs in *MSH6* or *PMS2* (*MSH6*: 18.6%, 26/140; *PMS2*: 29.0%, 36/124) compared to women with PVs in *MLH1* or *MSH2* (*MLH1*: 3.1%, 2/65; *MSH2*: 4.3%, 4/94) (*p* < 0.001) (Figure 1b).

The average numbers of reported breast, ovarian, endometrial, and colorectal cancers per family showed similar results to personal cancer histories (Figure 1c). Breast cancer was more commonly reported in family members (up to third degree as available) of probands with a PV in *MSH6* or *PMS2* (average number of reported cancers per family = 1.46 and 1.52) whereas colon cancer was more commonly reported in family members of probands with a PV in *MLH1* or *MSH2* (2.62 and 2.12). The proportion of probands (excluding the seven relatives in our cohort) reporting a personal or family history of breast cancer but no colorectal, endometrial, or ovarian cancer was lower among those with *MLH1* or *MSH2* PVs (*MLH1*: 0%, 0/65; *MSH2*: 2.2%, 2/92) than among those with PVs in *MSH6* or *PMS2* (*MSH6*: 8.0%, 11/137; *PMS2*: 24.6%, 30/122) (*p* < 0.001).

### SIR analysis and cumulative risk

Breast cancer risk was found to be approximately twofold higher (SIR = 1.96; 95% CI, 1.63–2.37) in our aggregate mismatch repair PV cohort compared to the general population (Table 2). When evaluating by gene, only two of the four MMR genes were found to have a statistically significant increased association with breast cancer: *MSH6* (SIR = 2.11; 95% CI, 1.56–2.86) and *PMS2* (SIR = 2.92; 95% CI, 2.17–3.92). *MLH1* (SIR = 0.87; 95% CI, 0.416–1.83) and *MSH2* (SIR = 1.22; 95% CI, 0.721–2.06) were not found to be associated with breast cancer.

**Table 2:** Standard incidence ratios by MMR gene

	*MLH1*(*N*** = 64**^^^)	*MSH2*(*N*** = 94)**	*MSH6*(*N*** = 140)**	*PMS2*(*N*** = 124)**	**Total (** *N* ** = 422)**
**Breast Cancer**					
Observed, No.	7	14	42	44	107
Expected, No.	8.01	11.50	19.87	15.09	54.48
SIRs	0.87	1.22	2.11	2.92	1.96
95% CI	(0.43–1.83)	(0.72–2.06)	(1.56–2.86)	(2.17–3.92)	(1.63–2.37)
*p* value	0.72	0.46	<0.001	<0.001	<0.001
**Colon Cancer**					
Observed, No.	34	33	19	20^*^	106
Expected, No.	1.74	2.49	6.01	4.30	14.54
SIRs	19.53	13.25	3.16	4.65	7.29
95% CI	(13.96–27.34)	(9.42–18.64)	(2.02–4.96)	(2.30–7.20)	(6.03–8.82)
*p* value	<0.001	<0.001	<0.001	<0.001	<0.001
**Endometrial Cancer**					
Observed, No.	17	32	59	20	128
Expected, No.	1.31	1.79	3.84	2.96	9.90
SIRs	12.97	17.90	15.35	6.76	12.93
95% CI	(8.06–20.86)	(12.66–25.32)	(11.89–19.81)	(4.36–10.47)	(10.87–15.37)
*p* value	<0.001	<0.001	<0.001	<0.001	<0.001
**Ovarian Cancer**					
Observed, No.	6	13	21	4	44
Expected, No.	0.79	1.08	1.98	1.55	5.40
SIRs	7.57	12.07	10.62	2.58	8.15
95% CI	(3.40–16.86)	(7.01–20.78)	(6.92–16.26)	(0.97–6.87)	(6.07–10.95)
*p* value	<0.001	<0.001	<0.001	0.049	<0.001

CI, confidence interval; MMR, mismatch repair; no., number; SIR, standard incidence ratio.

^^^One individual with an *MLH1* PV was removed from all SIR calculations as she was only 18 at the time of testing and Surveillance, Epidemiology, and End Results incidence rates do not exist for individuals <20 years of age.

^*^One individual with a *PMS2* PV was removed from the colon cancer SIR calculation as her colon cancer history was ambiguous.

**Figure 2:**
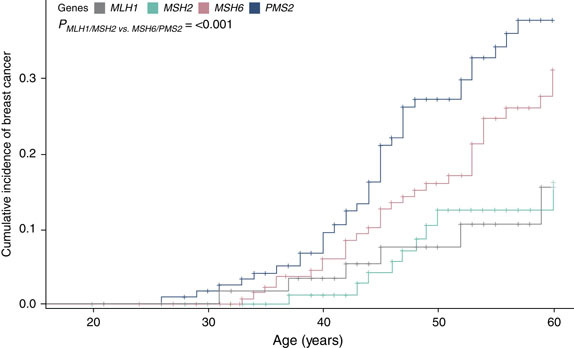
Cumulative incidence of breast cancer. All study participants with breast cancer and a prior cancer diagnosis were assessed to see if there were any potential treatment-related risks that could have contributed to their breast cancer. It was determined that no study participants needed to be excluded based on previous treatment.

Cumulative incidence of breast cancer was calculated and illustrated via Kaplan–Meier analysis (Figure 2). The cumulative incidence of breast cancer at the age of 60 was found to be 37.7% (95% CI, 27.5–47.8) for *PMS2*, 31.1% (95% CI, 21.9–40.7) for *MSH6*, 16.1% (95% CI, 7.3–27.9) for *MSH2*, and 15.5% (95% CI, 5.5–30.2) for *MLH1* PV carriers. *MSH6* and *PMS2* had a statistically significant greater cumulative incidence of breast cancer compared with *MLH1* and *MSH2* (*p* < 0.001).

### Clinical criteria

**Figure 3:**
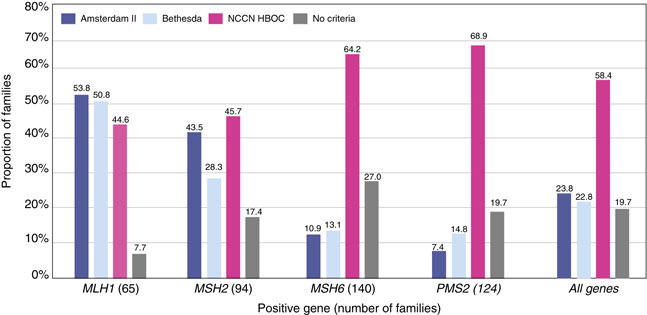
Clinical criteria. Each family is only counted once. Total families = 416. NCCN HBOC, National Comprehensive Cancer Network Hereditary Breast and Ovarian Cancer.

Unrelated probands (*n* = 416) were evaluated to determine if they met Amsterdam II, Revised Bethesda Guidelines, and the National Comprehensive Cancer Network (NCCN) *BRCA1/2* Testing Criteria (Figure 3).^33,34,35^ Women with *MLH1* (53.8%, 35/65) or *MSH2* (43.5%, 40/92) PVs, compared with *MSH6* and *PMS2*, were more likely to meet Amsterdam II criteria. Women with *MLH1* PVs most frequently met Revised Bethesda Guidelines (50.8%, 33/65). A higher proportion of women with *MSH6* (64.2%, 88/137) or *PMS2* (68.9%, 84/122) PVs met NCCN *BRCA1*/2 testing criteria compared to those with *MLH1* (44.6%, 29/65) or *MSH2* (45.7%, 42/92) PVs. Overall, our cohort of women with MMR PVs more commonly met NCCN *BRCA1/2* testing criteria (58.4%, 243/416) than any of the established LS clinical or testing criteria (Amsterdam II: 23.8%, 99/416; Revised Bethesda: 22.8%, 95/416).

## Discussion

We describe the reported personal and family cancer histories for a consecutive series of women identified to carry a single germ-line MMR PV through clinical testing with hereditary cancer gene panels. This study is the first, to our knowledge, to provide gene-specific breast cancer risks for all four MMR genes utilizing the same study cohort. In addition, unlike many previous studies, our cohort was not ascertained based on strict LS clinical criteria, suggesting our study population may be more representative of the population undergoing hereditary cancer testing. While many women had a personal or family history of traditional cancers associated with LS (colorectal, endometrial, or ovarian), 11.1% (46/416) reported no personal or family history of these cancers. In the absence of large panel testing, many might not have been tested for the MMR genes. In addition, three women with breast cancer were found to be positive for a MMR gene PV only after having normal results on a smaller, high-risk breast panel.

We found a twofold and threefold increased risk of breast cancer for the women with *MSH6* and *PMS2* PVs in our cohort, whereas no breast cancer association was observed for *MLH1* or *MSH2*. PVs in *MSH6* and *PMS2* were found to confer 31.1% and 37.7% cumulative risks for breast cancer by the age of 60 while PVs in *MLH1* or *MSH2* were found to confer breast cancer risks close to the expected general population risk, 16.1% and 15.5%, respectively.

Due to differences in study design, cohort ascertainment, and overall proportion of *MSH6* and *PMS2* PV carriers, many previous studies evaluating breast cancer risk in association with the MMR genes are not suitable for direct comparison with our findings.^15,16^ In one retrospective study with a design more similar to ours, Engel et al.^21^ found an almost twofold increased breast cancer risk in a cohort of 1,107 women with PVs in the MMR genes (SIR = 1.9; 95% CI, 1.4–2.4). Although Engel’s study did not include any women with *PMS2* PVs and included a smaller proportion of women with *MSH6* PVs (16.4%, C. Engel, personal communication) than the current study (33.1%), our SIR of 1.96 for all four MMR genes was similar to theirs.

As a clinical laboratory, it was important to determine whether our calculated SIRs for breast cancer might be skewed if our referral population were higher risk than the general population. We therefore calculated SIRs for well-established female-specific LS-associated cancers (endometrial and ovarian) using the same cohort of 423 women with MMR PVs. In aggregate, the endometrial cancer risk was found to be 13-fold higher (SIR = 12.93; 95% CI, 10.87–15.37) while the ovarian cancer risk was eightfold higher (SIR = 8.15; 95% CI, 6.61–10.95) compared with the general population, consistent with published LS-associated endometrial (SIRs = 10–62) and ovarian (SIRs = 7–14) cancer risks.^36^ As LS study cohorts in the literature have historically been ascertained using established clinical and/or testing criteria, resulting in overestimation of published CRC risks, we did not use CRC risks as a metric for comparison.^37^ The fact that the endometrial and ovarian SIRs in our cohort are consistent with previously published SIRs supports the validity of our approach.

Our findings suggest that the current LS clinical testing criteria may not be sufficiently sensitive, particularly for *MSH6* and *PMS2*. Hegde et al.^38^ reported that *MSH6* and *PMS2* account for approximately 7–10% and <5%, respectively, of LS families. When applying Amsterdam II criteria to our cohort, consistent with Hedge et al., only 10.9% of women with *MSH6* and 7.4% of *PMS2* PVs would have been identified. The overall low proportion of women with MMR PVs meeting Amsterdam II Criteria (23.9%), compared with those meeting NCCN *BRCA1/2* testing criteria (58.2%), underscores the need to continuously reconsider the criteria for which genes to test.

This study has several limitations. First, the clinical information used in this study was limited to that provided with the testing sample. In addition, the study population consisted only of women considered candidates for genetic testing by their providers and may not be representative of all women with MMR PVs. As our overall testing cohort was comprised largely of women with a personal or family history of breast cancer, SIRs might be biased and will need to be confirmed in other studies. It is notable that the race/ethnicity distribution between this study cohort and the general population differs slightly among certain groups.^39^ To address these potential differences we conducted sensitivity analyses limited to those reported to be only Caucasian/European (non-Hispanic white) in our cohort using breast cancer SEER rates for non-Hispanic white females and found similar results (results not shown). Finally, given our retrospective study design, our estimates of cumulative survival may be overestimated because we may have differentially tested women with longer survival. It is also possible that the retrospective design and young average age at time of testing (52.1 years in this study) underestimates the cumulative incidence. To deal with this latter bias, we estimated cumulative incidence to age 60 only. Prospective validation of our findings will be needed to minimize these and other potential biases.

This study suggests that women with PVs in *MSH6* or *PMS2* may benefit from increased breast cancer screening. Replication of our gene-specific breast cancer risks will be important to the future development of breast cancer management guidelines for female LS patients. In addition, further assessment of MMR deficiency in breast tumors may provide more information with which to evaluate the link between MMR genes and breast cancer. Finally, family studies of additional individuals with MMR PVs may also provide better breast cancer risk estimates.

In conclusion, our data demonstrate that PVs in *MSH6* or *PMS2* are associated with a modest but statistically significant increased risk for breast cancer. Previously, the identification of a PV in *MSH6* or *PMS2* in a woman with breast cancer may have been considered an incidental finding. Here we show that PVs in *MSH6* or *PMS2* are associated with breast cancer and that these MMR genes should be considered when ordering a multigene panel for women with a personal or family history of breast cancer.

## Electronic supplementary material

Supplementary Tables

Supplementary Figure 1

## Electronic supplementary material

**Supplementary material** is linked to the online version of the paper at 10.1038/gim.2017.254
