# Supplementary material for: MSH6 and PMS2 germ-line pathogenic variants implicated in Lynch syndrome are associated with breast cancer
Source: Genet Med. 2018 Jan 18;20(10):1167–74. doi: 10.1038/gim.2017.254 (PMC6051923; doi:10.1038/gim.2017.254)
Supplement: Supplementary file 1 — Supplementary Tables [file 41436_2018_201_MOESM1_ESM.docx]

**Supplemental Table 1. Inherited Cancer Panels and Breast Cancer History among 423 Women with PVs in MMR Genes**

| Cancer Category | Panel | No. women tested with each panel (% of total cohort) | No. women (%) with breast cancer of those tested with that panel | Genes on Panel^ |
| --- | --- | --- | --- | --- |
| General  (Includes Lynch and Breast Cancer Genes) | Comprehensive Cancer Panel | 104 (24.6%) | 27 (26.0%) | *APC; ATM; AXIN2; BARD1; BMPR1A; BRCA1; BRCA2; BRIP1; CDH1; CDK4; CDKN2A; CHEK2; EPCAM; FANCC; GREM1; MLH1; MSH2; MSH6; MUTYH; NBN; PALB2; PMS2; POLD1; POLE; PTEN; RAD51C; RAD51D; SCG5/GREM1; SMAD4; STK11; TP53; VHL; XRCC2* |
|  | High/Moderate Risk Panel | 52 (12.3%) | 21 (40.4%) | *APC; ATM; BMPR1A; BRCA1; BRCA2; BRIP1; CDH1; CDKN2A; CHEK2; EPCAM; MLH1; MSH2; MSH6; MUTYH; PALB2; PMS2; PTEN; RAD51C; RAD51D; SMAD4; STK11; TP53; VHL* |
| Breast and Ovarian | Breast/Ovarian Cancer Panel | 103 (24.3%) | 47 (45.6%) | *ATM; BARD1; BRCA1; BRCA2; BRIP1; CDH1; CHEK2; EPCAM; FANCC; MLH1; MSH2; MSH6; NBN; PALB2; PMS2; PTEN; RAD51C; RAD51D; STK11; TP53; XRCC2* |
| Lynch/Colorectal | Lynch/Colorectal High Risk Panel | 64 (15.1%) | 2 (3.1%) | *APC; EPCAM; MLH1; MSH2; MSH6; MUTYH; PMS2* |
|  | Colorectal Cancer Panel | 47 (11.1%) | - | *APC; ATM; AXIN2; BMPR1A; CDH1; CHEK2; EPCAM; MLH1; MSH2; MSH6; MUTYH; PMS2; POLD1; POLE; PTEN; SCG5/GREM1; SMAD4; STK11; TP53* |
|  | Lynch Syndrome Panel | 6 (1.4%) | - | *EPCAM; MLH1; MSH2; MSH6; PMS2* |
| Endometrial | Endometrial Cancer Panel | 44 (10.4%) | 7 (15.9%) | *BRCA1; BRCA2; CHEK2; EPCAM; MLH1; MSH2; MSH6; MUTYH; PMS2; POLD1; PTEN; TP53* |
| Reflex panels after High Risk Breast | Reflex to Remainder of Cancer Panel after Breast Cancer High Risk Panel | 2 (0.5%) | 2 (100.0%) | *APC; ATM; AXIN2; BARD1; BMPR1A; BRIP1; CDK4; CDKN2A; CHEK2; EPCAM; FANCC; MLH1; MSH2; MSH6; MUTYH; NBN; PALB2; PMS2; POLD1; POLE; RAD51C; RAD51D; SCG5/GREM1; SMAD4; STK11; VHL; XRCC2* |
|  | Reflex to Remainder of Cancer Panel Breast Cancer High Risk Panel and PALB2 | 1 (0.2%) | 1 (100.0%) | *APC; ATM; AXIN2; BARD1; BMPR1A; BRIP1; CDK4; CDKN2A; CHEK2; EPCAM; FANCC; MLH1; MSH2; MSH6; MUTYH; NBN; PMS2; POLD1; POLE; RAD51C; RAD51D; SCG5/GREM1; SMAD4; STK11; VHL; XRCC2* |
|  | High Risk Breast Panel* | - | - | *BRCA1; BRCA2; CDH1; PTEN; STK11; TP53* |
| Total |  | **423 (100%)** | **107 (25.3%)** |  |

^Panel composition has changed over time. This gene list represents the genes included on each panel as of April 2017.

*The gene list for the High Risk Breast Panel is included for reference only. It does not contain the MMR genes.

**Supplemental Table 2. Pathogenic or Likely Pathogenic Variants in *MLH1*, *MSH2*, *MSH6* and *PMS2* Identified *in* 423 Women**

| Gene | Study ID | Cancer Panel | cDNA | Protein | Variant Type | Variant Classification | Personal Cancer History |
| --- | --- | --- | --- | --- | --- | --- | --- |
| *MLH1* | 29650 | Comprehensive | c.150_153delTGTT | p.Val51LysfsX5 | Frameshift | Pathogenic | Endometrial |
| *MLH1* | 5488 | Comprehensive | c.155_156delAA | p.Lys52ArgfsX26 | Frameshift | Pathogenic | Colorectal |
| *MLH1* | 39994 | Colorectal | c.199G>A | p.Gly67Arg | Missense | Pathogenic | Unaffected |
| *MLH1* | 33679 | Breast/Ovarian | c.200G>A | p.Gly67Glu | Missense | Pathogenic | Endometrial |
| *MLH1* | 3395 | Lynch/CRC High Risk | c.208-3C>G | IVS2-3 C>G | Splice | Likely Pathogenic | Colorectal |
| *MLH1* | 36161 | Breast/Ovarian | c.272T>G | p.Leu91Ter | Nonsense | Pathogenic | Endometrial; Gynecologic |
| *MLH1* | 43491 | Lynch/CRC High Risk | c.298C>T | p.Arg100Ter | Nonsense | Pathogenic | Colorectal |
| *MLH1* | 12334 | Endometrial | c.350C>T | p.Thr117Met | Missense | Pathogenic | Breast |
| *MLH1* | 6075 | Comprehensive | c.453+1G>T | IVS5+1 G>T | Splice | Pathogenic | Colorectal |
| *MLH1* | 2826 | Lynch/CRC High Risk | c.503dupA | p.Asn168LysfsX4 | Frameshift | Pathogenic | Colorectal |
| *MLH1* | 15856 | Lynch/CRC High Risk | c.531_532delGGinsAT | p.Glu178Ter | Nonsense | Pathogenic | Colorectal |
| *MLH1* | 32272 | High/Moderate | c.588+5G>A | IVS7+5 G>A | Splice | Pathogenic | Breast; Gastric |
| *MLH1* | 2778 | Endometrial | c.589-2A>G | IVS7-2 A>G | Splice | Pathogenic | Endometrial |
| *MLH1* | 8612 | Comprehensive | c.589-2A>G | IVS7-2A>G | Splice | Pathogenic | Colorectal |
| *MLH1* | 34071 | Colorectal | c.589-2A>G | IVS7-2A>G | Splice | Pathogenic | Colorectal |
| *MLH1* | 1522 | Lynch/CRC High Risk | c.676C>T | p.Arg226Ter | Nonsense | Pathogenic | Sebaceous Neoplasm |
| *MLH1* | 1715 | Endometrial | c.676C>T | p.Arg226Ter | Nonsense | Pathogenic | Breast; Colorectal |
| *MLH1* | 19113 | Lynch/CRC High Risk | c.676C>T | p.Arg226Ter | Nonsense | Pathogenic | Colorectal |
| *MLH1* | 22021 | Lynch/CRC High Risk | c.677+1G>T | IVS8+1 G>T | Splice | Pathogenic | Colorectal |
| *MLH1* | 21197 | Breast/Ovarian | c.677G>A | p.Arg226Gln | Cryptic Splice | Pathogenic | Unaffected |
| *MLH1* | 7491 | High/Moderate | c.677G>T | p.Arg226Leu | Cryptic Splice | Likely Pathogenic | Breast |
| *MLH1* | 11966 | Comprehensive | c.677G>T | p.Arg226Leu | Cryptic Splice | Likely Pathogenic | Colorectal |
| *MLH1* | 33080 | Colorectal | c.790+1G>A | IVS9+1 G>A | Splice | Pathogenic | Colorectal |
| *MLH1* | 39105 | Endometrial | c.791-2A>G | IVS9-2 A>G | Splice | Pathogenic | Unaffected |
| *MLH1* | 33156 | Endometrial | c.791-2A>G | IVS9-2 A>G | Splice | Pathogenic | Breast; Colorectal; Ovarian |
| *MLH1* | 22242 | Colorectal | c.791-2A>T | IVS9-2 A>T | Splice | Pathogenic | Colorectal |
| *MLH1* | 3612 | Lynch/CRC High Risk | c.971dupA | p.Arg325AlafsX37 | Frameshift | Pathogenic | Colorectal |
| *MLH1* | 16403 | Breast/Ovarian | c.1004delT | p.Leu335ArgfsX32 | Nonsense | Pathogenic | Appendiceal |
| *MLH1* | 11213 | Lynch/CRC High Risk | c.1011dupC | p.Asn338GlnfsX24 | Frameshift | Pathogenic | Unaffected |
| *MLH1* | 32668 | Breast/Ovarian | c.1029delC | p.Phe344SerfsX23 | Frameshift | Pathogenic | Unaffected |
| *MLH1* | 4332 | Colorectal | c.1039-1G>A | IVS11-1 G>A | Splice | Pathogenic | Colorectal; Endometrial |
| *MLH1* | 42730 | Colorectal | c.1105dupT | p.Ser369PhefsX7 | Frameshift | Pathogenic | Colorectal |
| *MLH1* | 28683 | Comprehensive | c.1381A>T | p.Lys461Ter | Nonsense | Pathogenic | Carcinoid Tumor; Ovarian |
| *MLH1* | 23822 | Lynch/CRC High Risk | c.1407dupC | p.Arg470GlnfsX9 | Frameshift | Pathogenic | Endometrial |
| *MLH1* | 10666 | Lynch/CRC High Risk | c.1459C>T | p.Arg487Ter | Nonsense | Pathogenic | Endometrial |
| *MLH1* | 14948 | Lynch/CRC High Risk | c.1459C>T | p.Arg487Ter | Nonsense | Pathogenic | Endometrial |
| *MLH1* | 43777 | Comprehensive | c.1459C>T | p.Arg487Ter | Nonsense | Pathogenic | Ovarian |
| *MLH1* | 5303 | Comprehensive | c.1489dupC | p.Arg497ProfsX6 | Frameshift | Pathogenic | Endometrial |
| *MLH1* | 7891 | Colorectal | c.1489dupC | p.Arg497ProfsX6 | Frameshift | Pathogenic | Colorectal |
| *MLH1* | 27185 | Lynch/CRC High Risk | c.1520_1521insTA | p.Leu507PhefsX29 | Frameshift | Likely Pathogenic | Endometrial |
| *MLH1* | 8402 | Endometrial | c.1559-1G>T | IVS13-1 G>T | Splice | Pathogenic | Colorectal; Endometrial |
| *MLH1* | 41716 | Colorectal | c.1637_1641dupAGTTA | p.Tyr548SerfsX45 | Frameshift | Pathogenic | Colorectal |
| *MLH1* | 7396 | Lynch/CRC High Risk | c.1731G>A | p.Ser577Ser | Cryptic Splice | Pathogenic | Colorectal |
| *MLH1* | 21208 | Lynch/CRC High Risk | c.1736_1737insGT | p.Ala580Ter | Nonsense | Pathogenic | Colorectal |
| *MLH1* | 4374 | Colorectal | c.1852_1854delAAG | p.Lys618del | In Frame | Pathogenic | Colorectal |
| *MLH1* | 4422 | Lynch/CRC High Risk | c.1852_1854delAAG | p.Lys618del | In Frame | Pathogenic | Colorectal |
| *MLH1* | 37505 | Comprehensive | c.1852_1854delAAG | p.Lys618del | In Frame | Pathogenic | Colorectal |
| *MLH1* | 16555 | Comprehensive | c.1897G>T | p.Glu633Ter | Nonsense | Pathogenic | Ovarian |
| *MLH1* | 31895 | Lynch/CRC High Risk | c.1961C>T | p.Pro654Leu | Missense | Pathogenic | Skin (SCC) |
| *MLH1* | 37265 | Breast/Ovarian | c.1961C>T | p.Pro654Leu | Missense | Pathogenic | Ampullary |
| *MLH1* | 34856 | Endometrial | c.1989G>C | p.Glu663Asp | Cryptic Splice | Likely Pathogenic | Bile Duct |
| *MLH1* | 39192 | Colorectal | c.1989G>T | p.Glu663Asp | Cryptic Splice | Pathogenic | Colorectal; Ovarian |
| *MLH1* | 4485 | Comprehensive | c.2065C>T | p.Gln689Ter | Nonsense | Pathogenic | Breast; Colorectal |
| *MLH1* | 16676 | Comprehensive | c.2206G>T | p.Glu736Ter | Nonsense | Pathogenic | Colorectal |
| *MLH1* | 38603 | Comprehensive | Deletion Exon 1 |  | Large Deletion | Pathogenic | Endometrial |
| *MLH1* | 864 | Breast/Ovarian | Deletion Exons 1-13 |  | Large Deletion | Pathogenic | Breast; Endometrial |
| *MLH1* | 6202 | High/Moderate | Deletion Exons 2-3 |  | Large Deletion | Pathogenic | Endometrial |
| *MLH1* | 31612 | Colorectal | Deletion Exon 3 |  | Large Deletion | Pathogenic | Colorectal; Endometrial |
| *MLH1* | 5531 | Colorectal | Duplication Exons 6-12 |  | Large Duplication | Pathogenic | Colorectal |
| *MLH1* | 3181 | Colorectal | Deletion Exon 16 |  | Large Deletion | Pathogenic | Colorectal |
| *MLH1* | 1740 | Comprehensive | Deletion Exons 16-19 |  | Large Deletion | Pathogenic | Endometrial; Ovarian |
| *MLH1* | 8762 | Colorectal | Deletion Exons 16-19 |  | Large Deletion | Pathogenic | Cervical |
| *MLH1* | 10965 | Endometrial | Deletion Exons 16-19 |  | Large Deletion | Pathogenic | Endometrial |
| *MLH1* | 32249 | Lynch/CRC High Risk | Deletion Exons 16-19 |  | Large Deletion | Pathogenic | Bladder; Colorectal; Endometrial |
| *MLH1* | 380 | Lynch/CRC High Risk | Deletion Exons 16-19 |  | Large Deletion | Pathogenic | Colorectal |
| *MSH2* | 859 | Breast/Ovarian | c.70C>T | p.Gln24Ter | Nonsense | Pathogenic | Endometrial; Primary Peritoneal |
| *MSH2* | 25529 | Colorectal | c.181C>T | p.Gln61Ter | Nonsense | Pathogenic | Bladder; Colorectal |
| *MSH2* | 24146 | Lynch/CRC High Risk | c.212-1G>A | IVS1-1 G>A | Splice | Pathogenic | Endometrial |
| *MSH2* | 11025 | Breast/Ovarian | c.387_388delTC | p.Gln130ValfsX2 | Frameshift | Pathogenic | Breast; Endometrial; Ovarian |
| *MSH2* | 35329 | High/Moderate | c.387_388delTC | p.Gln130ValfsX2 | Frameshift | Pathogenic | Endometrial |
| *MSH2* | 9182 | Colorectal | c.508C>T | p.Gln170Ter | Nonsense | Pathogenic | Colorectal; Endometrial |
| *MSH2* | 34664 | Lynch/CRC High Risk | c.508C>T | p.Gln170Ter | Nonsense | Pathogenic | Colorectal; Sebaceous Neoplasm; Skin (BCC) |
| *MSH2* | 10447 | High/Moderate | c.571_573delCTC | p.Leu191del | In Frame | Likely Pathogenic | Endometrial; Desmoid Tumor |
| *MSH2* | 25763 | Comprehensive | c.792+1delG | IVS4+1delG | Splice | Pathogenic | Unaffected |
| *MSH2* | 21525 | Comprehensive | c.806_807ins13 | p.Leu270ValfsX18 | Frameshift | Pathogenic | Colorectal; Endometrial |
| *MSH2* | 8329 | Comprehensive | c.862C>T | p.Gln288Ter | Nonsense | Pathogenic | Colorectal; Endometrial |
| *MSH2* | 1155 | Lynch/CRC High Risk | c.932delA | p.Asn311ThrfsX20 | Frameshift | Pathogenic | Unaffected |
| *MSH2* | 1720 | Lynch/CRC High Risk | c.942+3A>T | IVS5+3 A>T | Splice | Pathogenic | Ovarian |
| *MSH2* | 3419 | Lynch/CRC High Risk | c.942+3A>T | IVS5+3 A>T | Splice | Pathogenic | Colorectal |
| *MSH2* | 3688 | Lynch/CRC High Risk | c.942+3A>T | IVS5+3 A>T | Splice | Pathogenic | Bladder; Colorectal |
| *MSH2* | 8786 | Breast/Ovarian | c.942+3A>T | IVS5+3 A>T | Splice | Pathogenic | Unaffected |
| *MSH2* | 24026 | High/Moderate | c.942+3A>T | IVS5+3 A>T | Splice | Pathogenic | Breast; Colorectal |
| *MSH2* | 32985 | High/Moderate | c.942+3A>T | IVS5+3 A>T | Splice | Pathogenic | Breast; Ovarian |
| *MSH2* | 36667 | High/Moderate | c.942+3A>T | IVS5+3 A>T | Splice | Pathogenic | Breast; Ovarian |
| *MSH2* | 37970 | Comprehensive | c.942+3A>T | IVS5+3 A>T | Splice | Pathogenic | Sebaceous Neoplasm |
| *MSH2* | 25378 | Colorectal | c.942+3A>T | IVS5+3A>T | Splice | Pathogenic | Endometrial |
| *MSH2* | 7496 | Comprehensive | c.998G>A | p.Cys333Tyr | Missense | Pathogenic | Breast; Ovarian |
| *MSH2* | 31752 | Comprehensive | c.998G>A | p.Cys333Tyr | Missense | Pathogenic | Colorectal |
| *MSH2* | 34444 | Colorectal | c.998G>A | p.Cys333Tyr | Missense | Pathogenic | Colorectal |
| *MSH2* | 14899 | Colorectal | c.1008delT | p.Gln337LysfsX20 | Frameshift | Pathogenic | Colorectal; Endometrial; Renal |
| *MSH2* | 40074 | Comprehensive | c.1023delT | p.Val342LeufsX15 | Frameshift | Pathogenic | Colorectal |
| *MSH2* | 40678 | Comprehensive | c.1076+1G>A | IVS6+1 G>A | Splice | Pathogenic | Colorectal |
| *MSH2* | 4363 | Endometrial | c.1157dupA | p.Asp386GlufsX3 | Frameshift | Pathogenic | Endometrial |
| *MSH2* | 37699 | Breast/Ovarian | c.1158_1167del10 | p.Asn388ProfsX21 | Frameshift | Pathogenic | Unaffected |
| *MSH2* | 41057 | Breast/Ovarian | c.1158_1167del10 | p.Asn388ProfsX21 | Frameshift | Pathogenic | Unaffected |
| *MSH2* | 20303 | Comprehensive | c.1165C>T | p.Arg389Ter | Nonsense | Pathogenic | Unaffected |
| *MSH2* | 27095 | Comprehensive | c.1165C>T | p.Arg389Ter | Nonsense | Pathogenic | Breast |
| *MSH2* | 43642 | Comprehensive | c.1165C>T | p.Arg389Ter | Nonsense | Pathogenic | Unaffected |
| *MSH2* | 6545 | Lynch/CRC High Risk | c.1226_1227delAG | p.Gln409ArgfsX7 | Frameshift | Pathogenic | Endometrial |
| *MSH2* | 28629 | High/Moderate | c.1226_1227delAG | p.Gln409ArgfsX7 | Frameshift | Pathogenic | Ovarian |
| *MSH2* | 34496 | Colorectal | c.1226_1227delAG | p.Gln409ArgfsX7 | Frameshift | Pathogenic | Anal |
| *MSH2* | 14769 | Endometrial | c.1276+2T>C | IVS7+2 T>C | Splice | Pathogenic | Breast; Endometrial |
| *MSH2* | 33578 | Comprehensive | c.1276+2T>C | IVS7+2 T>C | Splice | Pathogenic | Colorectal; Endometrial |
| *MSH2* | 4004 | Comprehensive | c.1373T>G | p.Leu458Ter | Nonsense | Pathogenic | Breast |
| *MSH2* | 29663 | Lynch/CRC High Risk | c.1457_1460delATGA | p.Asn486ThrfsX10 | Frameshift | Pathogenic | Colorectal; Sarcoma |
| *MSH2* | 7627 | Endometrial | c.1525A>T | p.Lys509Ter | Nonsense | Pathogenic | Endometrial; Ovarian |
| *MSH2* | 30854 | High/Moderate | c.1552_1553delCA | p.Gln518ValfsX10 | Frameshift | Pathogenic | Breast |
| *MSH2* | 14826 | Breast/Ovarian | c.1552C>T | p.Gln518Ter | Nonsense | Pathogenic | Ovarian |
| *MSH2* | 43073 | Colorectal | c.1571G>C | p.Arg524Pro | Missense | Likely Pathogenic | Colorectal; Endometrial |
| *MSH2* | 43144 | Comprehensive | c.1576delA | p.Thr526ProfsX17 | Frameshift | Pathogenic | Breast; Colorectal; Endometrial |
| *MSH2* | 42380 | Breast/Ovarian | c.1738G>T | p.Glu580Ter | Nonsense | Pathogenic | Endometrial; Ovarian |
| *MSH2* | 4908 | Lynch/CRC High Risk | c.1786_1788delAAT | p.Asn596del | In Frame | Pathogenic | Endometrial |
| *MSH2* | 44231 | Endometrial | c.1786_1788delAAT | p.Asn596del | In Frame | Pathogenic | Brain; Endometrial |
| *MSH2* | 15861 | High/Moderate | c.1801C>T | p.Gln601Ter | Nonsense | Pathogenic | Breast |
| *MSH2* | 15058 | High/Moderate | c.1861C>T | p.Arg621Ter | Nonsense | Pathogenic | Colorectal; Renal; Sebaceous Neoplasm |
| *MSH2* | 17873 | Endometrial | c.1861C>T | p.Arg621Ter | Nonsense | Pathogenic | Endometrial |
| *MSH2* | 25429 | High/Moderate | c.1861C>T | p.Arg621Ter | Nonsense | Pathogenic | Unaffected |
| *MSH2* | 43454 | Comprehensive | c.1883delG | p.Gly628AspfsX7 | Frameshift | Pathogenic | Breast; Endometrial |
| *MSH2* | 4974 | Lynch/CRC High Risk | c.1906G>C | p.Ala636Pro | Missense | Pathogenic | Colorectal; Thyroid |
| *MSH2* | 6735 | Lynch/CRC High Risk | c.1916_1919delATGC | p.His639LeufsX45 | Frameshift | Pathogenic | Colorectal |
| *MSH2* | 41912 | Lynch/CRC High Risk | c.1984C>T | p.Gln662Ter | Nonsense | Pathogenic | Sebaceous Neoplasm |
| *MSH2* | 25058 | High/Moderate | c.2021G>A | p.Gly674Asp | Missense | Likely Pathogenic | Unaffected |
| *MSH2* | 18563 | Lynch/CRC High Risk | c.2027C>G | p.Ser676Ter | Nonsense | Pathogenic | Colorectal |
| *MSH2* | 2524 | Breast/Ovarian | c.2038C>T | p.Arg680Ter | Nonsense | Pathogenic | Sebaceous Neoplasm |
| *MSH2* | 4361 | Colorectal | c.2038C>T | p.Arg680Ter | Nonsense | Pathogenic | Colorectal; Skin (SCC) |
| *MSH2* | 13107 | Endometrial | c.2038C>T | p.Arg680Ter | Nonsense | Pathogenic | Ovarian |
| *MSH2* | 27967 | Breast/Ovarian | c.2038C>T | p.Arg680Ter | Nonsense | Pathogenic | Breast; Pancreatic |
| *MSH2* | 27981 | Colorectal | c.2038C>T | p.Arg680Ter | Nonsense | Pathogenic | Unaffected |
| *MSH2* | 42842 | Colorectal | c.2038C>T | p.Arg680Ter | Nonsense | Pathogenic | Colorectal |
| *MSH2* | 20729 | Endometrial | c.2105T>A | p.Val702Glu | Missense | Likely Pathogenic | Unaffected |
| *MSH2* | 35944 | Breast/Ovarian | c.2228C>A | p.Ser743Ter | Nonsense | Pathogenic | Colorectal |
| *MSH2* | 22535 | Lynch/CRC High Risk | c.2459-2A>G | IVS14-2 A>G | Splice | Pathogenic | Colorectal; Endometrial |
| *MSH2* | 6633 | Breast/Ovarian | c.2647delA | p.Ile883LeufsX9 | Frameshift | Pathogenic | Ovarian |
| *MSH2* | 8154 | Breast/Ovarian | c.2647delA | p.Ile883LeufsX9 | Frameshift | Pathogenic | Unaffected |
| *MSH2* | 5712 | Breast/Ovarian | Deletion Exons 1-2 |  | Large Deletion | Pathogenic | Unaffected |
| *MSH2* | 35315 | Comprehensive | Deletion Exons 1-2 |  | Large Deletion | Pathogenic | Endometrial |
| *MSH2* | 898 | Lynch/CRC High Risk | Deletion Exons 1-6 |  | Large Deletion | Pathogenic | Endometrial; Ovarian |
| *MSH2* | 4730 | Comprehensive | Deletion Exons 1-6 |  | Large Deletion | Pathogenic | Breast; Colorectal; Cervical; Melanoma |
| *MSH2* | 5580 | Colorectal | Deletion Exons 1-6 |  | Large Deletion | Pathogenic | Colorectal |
| *MSH2* | 7008 | Colorectal | Deletion Exons 1-6 |  | Large Deletion | Pathogenic | Endometrial |
| *MSH2* | 20695 | Lynch/CRC High Risk | Deletion Exons 1-6 |  | Large Deletion | Pathogenic | Sebaceous Neoplasm; Gastric |
| *MSH2* | 21518 | Lynch Syndrome | Deletion Exons 1-6 |  | Large Deletion | Pathogenic | Unaffected |
| *MSH2* | 28059 | Lynch/CRC High Risk | Deletion Exons 1-6 |  | Large Deletion | Pathogenic | Endometrial; Colorectal; Skin (SCC) |
| *MSH2* | 31459 | Endometrial | Deletion Exons 1-6 |  | Large Deletion | Pathogenic | Endometrial |
| *MSH2* | 32184 | High/Moderate | Deletion Exons 1-6 |  | Large Deletion | Pathogenic | Endometrial |
| *MSH2* | 34804 | Colorectal | Deletion Exons 1-6 |  | Large Deletion | Pathogenic | Unaffected |
| *MSH2* | 37236 | Colorectal | Deletion Exons 1-6 |  | Large Deletion | Pathogenic | Sebaceous Neoplasm; Skin (BCC) |
| *MSH2* | 36362 | Colorectal | Deletion Exons 2-6 |  | Large Deletion | Pathogenic | Colorectal |
| *MSH2* | 15187 | Lynch/CRC High Risk | Deletion Exons 2-7 |  | Large Deletion | Pathogenic | Colorectal; Skin, NOS; Ureter |
| *MSH2* | 1472 | Colorectal | Deletion Exons 3-8 |  | Large Deletion | Pathogenic | Endometrial |
| *MSH2* | 32218 | Lynch/CRC High Risk | Deletion Exon 6 |  | Large Deletion | Pathogenic | Endometrial |
| *MSH2* | 20022 | Comprehensive | Deletion Exon 8 |  | Large Deletion | Pathogenic | Multiple Myeloma; Colorectal; Gastric |
| *MSH2* | 34241 | High/Moderate | Deletion Exon 9 |  | Large Deletion | Pathogenic | Endometrial |
| *MSH2* | 17835 | Colorectal | Deletion Exons 12-16 |  | Large Deletion | Pathogenic | Colorectal |
| *MSH2* | 41593 | Breast/Ovarian | Deletion Exons 15-16 |  | Large Deletion | Pathogenic | Unaffected |
| *MSH2* | 30499 | Colorectal | Deletion Exons 1-9 of EPCAM and Exons 1-3 of MSH2 |  | Large Deletion | Pathogenic | Endometrial; Sebaceous Neoplasm; Skin (BCC) |
| *MSH2* | 46702 | Colorectal | Deletion Exons 1-9 of EPCAM and Exons 1-6 of MSH2 |  | Large Deletion | Pathogenic | Unaffected |
| *MSH2* | 46703 | Lynch/CRC High Risk | Deletion Exons 1-9 of EPCAM and Exons 1-6 of MSH2 |  | Large Deletion | Pathogenic | Unaffected |
| *MSH2* | 5435 | Lynch/CRC High Risk | Deletion Exons 1-9 of EPCAM and Exons 1-7 of MSH2 |  | Large Deletion | Pathogenic | Colorectal |
| *MSH6* | 25818 | Breast/Ovarian | c.3G>T | p.Met1? | Start Codon | Likely Pathogenic | Breast |
| *MSH6* | 36247 | Breast/Ovarian | c.10C>T | p.Gln4Ter | Nonsense | Pathogenic | Ovarian |
| *MSH6* | 9848 | Comprehensive | c.255delC | p.Thr86ProfsX63 | Frameshift | Pathogenic | Endometrial |
| *MSH6* | 24096 | Breast/Ovarian | c.260+2_260+3delTAinsAG | IVS1+2_IVS1+3delTAinsAG | Splice | Pathogenic | Breast |
| *MSH6* | 29246 | High/Moderate | c.457+1delG | IVS2+1delG | Splice | Likely Pathogenic | Breast |
| *MSH6* | 14344 | Endometrial | c.467C>G | p.Ser156Ter | Nonsense | Pathogenic | Endometrial |
| *MSH6* | 35032 | Comprehensive | c.467C>G | p.Ser156Ter | Nonsense | Pathogenic | Unaffected |
| *MSH6* | 651 | Endometrial | c.468_471delAAAG | p.Glu158ProfsX15 | Frameshift | Pathogenic | Endometrial; Ovarian |
| *MSH6* | 35291 | Comprehensive | c.468_471delAAAG | p.Glu158ProfsX15 | Frameshift | Pathogenic | Endometrial |
| *MSH6* | 38460 | Endometrial | c.578delT | p.Leu193TrpfsX18 | Frameshift | Pathogenic | Unaffected |
| *MSH6* | 29673 | Colorectal | c.599C>G | p.Ser200Ter | Nonsense | Pathogenic | Endometrial |
| *MSH6* | 4262 | Comprehensive | c.702_703insT | p.Thr235TyrfsX5 | Frameshift | Pathogenic | Colorectal |
| *MSH6* | 44031 | Breast/Ovarian | c.817G>T | p.Gly273Ter | Nonsense | Pathogenic | Unaffected |
| *MSH6* | 20481 | High/Moderate | c.853dupA | p.Ser285LysfsX2 | Frameshift | Pathogenic | Breast; Endometrial |
| *MSH6* | 3191 | Comprehensive | c.892C>T | p.Arg298Ter | Nonsense | Pathogenic | Breast; Endometrial |
| *MSH6* | 19780 | High/Moderate | c.892C>T | p.Arg298Ter | Nonsense | Pathogenic | Endometrial |
| *MSH6* | 8339 | High/Moderate | c.952_962del11 | p.Glu318SerfsX7 | Frameshift | Pathogenic | Breast |
| *MSH6* | 15889 | Breast/Ovarian | c.1059dupT | p.Gly354TrpfsX4 | Frameshift | Pathogenic | Unaffected |
| *MSH6* | 4007 | Comprehensive | c.1135_1139delAGAGA | p.Arg379Ter | Nonsense | Pathogenic | Cervical |
| *MSH6* | 17261 | High/Moderate | c.1189dupT | p.Tyr397LeufsX4 | Frameshift | Pathogenic | Colorectal; Endometrial |
| *MSH6* | 7791 | Endometrial | c.1190_1191delAT | p.Tyr397CysfsX3 | Frameshift | Pathogenic | Endometrial |
| *MSH6* | 2739 | Endometrial | c.1241G>A | p.Trp414Ter | Nonsense | Pathogenic | Endometrial; Ovarian |
| *MSH6* | 40434 | Breast/Ovarian | c.1241G>A | p.Trp414Ter | Nonsense | Pathogenic | Endometrial; Ovarian |
| *MSH6* | 35501 | Endometrial | c.1255_1268del14 | p.Gln419CysfsX11 | Frameshift | Pathogenic | Bladder; Colorectal; Ovarian |
| *MSH6* | 33653 | Lynch/CRC High Risk | c.1304T>C | p.Leu435Pro | Missense | Likely Pathogenic | Breast; Endometrial |
| *MSH6* | 25902 | Endometrial | c.1367G>A | p.Trp456Ter | Nonsense | Pathogenic | Breast; Endometrial |
| *MSH6* | 10353 | Breast/Ovarian | c.1444C>T | p.Arg482Ter | Nonsense | Pathogenic | Breast |
| *MSH6* | 18034 | High/Moderate | c.1444C>T | p.Arg482Ter | Nonsense | Pathogenic | Endometrial |
| *MSH6* | 38009 | Endometrial | c.1444C>T | p.Arg482Ter | Nonsense | Pathogenic | Endometrial; Ovarian |
| *MSH6* | 18702 | Breast/Ovarian | c.1502_1511del10 | p.His501ArgfsX6 | Frameshift | Pathogenic | Ovarian |
| *MSH6* | 43243 | Comprehensive | c.1571dupA | p.Tyr524Ter | Nonsense | Pathogenic | Breast; Endometrial |
| *MSH6* | 2686 | Comprehensive | c.1634_1637delAAGA | p.Lys545ArgfsX25 | Frameshift | Pathogenic | Endometrial |
| *MSH6* | 3707 | Breast/Ovarian | c.1634_1637delAAGA | p.Lys545ArgfsX25 | Frameshift | Pathogenic | Breast |
| *MSH6* | 16346 | Colorectal | c.1645delT | p.Ser549LeufsX22 | Frameshift | Pathogenic | Colorectal |
| *MSH6* | 7760 | Breast/Ovarian | c.1805C>G | p.Ser602Ter | Nonsense | Pathogenic | Brain; Renal; Sarcoma |
| *MSH6* | 16945 | Breast/Ovarian | c.1819dupA | p.Thr607AsnfsX33 | Frameshift | Pathogenic | Ovarian |
| *MSH6* | 24752 | High/Moderate | c.1969C>T | p.Gln657Ter | Nonsense | Pathogenic | Endometrial |
| *MSH6* | 33150 | Breast/Ovarian | c.1969delC | p.Gln657ArgfsX6 | Frameshift | Pathogenic | Breast; Thyroid |
| *MSH6* | 11457 | Comprehensive | c.2057G>A | p.Gly686Asp | Missense | Likely Pathogenic | Sebaceous Neoplasm |
| *MSH6* | 26988 | Breast/Ovarian | c.2057G>A | p.Gly686Asp | Missense | Likely Pathogenic | Unaffected |
| *MSH6* | 29980 | Endometrial | c.2057G>A | p.Gly686Asp | Missense | Likely Pathogenic | Endometrial |
| *MSH6* | 9522 | Breast/Ovarian | c.2061T>A | p.Cys687Ter | Nonsense | Pathogenic | Breast; Colorectal; Endometrial |
| *MSH6* | 12668 | High/Moderate | c.2150_2153delTCAG | p.Val717AlafsX18 | Frameshift | Pathogenic | Colorectal |
| *MSH6* | 18881 | Breast/Ovarian | c.2150_2153delTCAG | p.Val717AlafsX18 | Frameshift | Pathogenic | Ovarian |
| *MSH6* | 43373 | Comprehensive | c.2150_2153delTCAG | p.Val717AlafsX18 | Frameshift | Pathogenic | Unaffected |
| *MSH6* | 3911 | Lynch/CRC High Risk | c.2194C>T | p.Arg732Ter | Nonsense | Pathogenic | Endometrial |
| *MSH6* | 16786 | Breast/Ovarian | c.2194C>T | p.Arg732Ter | Nonsense | Pathogenic | Breast |
| *MSH6* | 23993 | Breast/Ovarian | c.2194C>T | p.Arg732Ter | Nonsense | Pathogenic | Breast |
| *MSH6* | 43448 | Lynch/CRC High Risk | c.2194C>T | p.Arg732Ter | Nonsense | Pathogenic | Colorectal |
| *MSH6* | 19858 | Breast/Ovarian | c.2230dupG | p.Glu744GlyfsX12 | Frameshift | Pathogenic | Breast |
| *MSH6* | 15320 | High/Moderate | c.2269_2270delAC | p.Thr757ProfsX6 | Frameshift | Pathogenic | Endometrial; Ovarian |
| *MSH6* | 33779 | Lynch/CRC High Risk | c.2269_2270delAC | p.Thr757ProfsX6 | Frameshift | Pathogenic | Endometrial |
| *MSH6* | 29840 | Breast/Ovarian | c.2314C>T | p.Arg772Trp | Missense | Likely Pathogenic | Ovarian |
| *MSH6* | 26122 | Endometrial | c.2500_2501delAG | p.Gln835GlufsX11 | Frameshift | Pathogenic | Ovarian |
| *MSH6* | 44033 | Breast/Ovarian | c.2690dupA | p.Asn897LysfsX3 | Frameshift | Pathogenic | Breast |
| *MSH6* | 12666 | Colorectal | c.2731C>T | p.Arg911Ter | Nonsense | Pathogenic | Colorectal |
| *MSH6* | 29147 | Comprehensive | c.2731C>T | p.Arg911Ter | Nonsense | Pathogenic | Unaffected |
| *MSH6* | 20258 | Endometrial | c.2779dupA | p.Ile927AsnfsX8 | Frameshift | Pathogenic | Endometrial |
| *MSH6* | 4874 | Endometrial | c.2832_2833delAA | p.Ile944MetfsX4 | Frameshift | Pathogenic | Endometrial |
| *MSH6* | 39506 | Comprehensive | c.2832_2833delAA | p.Ile944MetfsX4 | Frameshift | Pathogenic | Breast |
| *MSH6* | 41370 | Lynch/CRC High Risk | c.2989A>T | p.Lys997Ter | Nonsense | Pathogenic | Unaffected |
| *MSH6* | 28596 | Breast/Ovarian | c.3013C>T | p.Arg1005Ter | Nonsense | Pathogenic | Breast |
| *MSH6* | 34009 | Comprehensive | c.3013C>T | p.Arg1005Ter | Nonsense | Pathogenic | Unaffected |
| *MSH6* | 8171 | Breast/Ovarian | c.3037_3041delAAGAA | p.Lys1013ValfsX3 | Frameshift | Pathogenic | Breast |
| *MSH6* | 1564 | Comprehensive | c.3155_3156delAG | p.Glu1052ValfsX13 | Frameshift | Pathogenic | Breast |
| *MSH6* | 5291 | Endometrial | c.3155_3156delAG | p.Glu1052ValfsX13 | Frameshift | Pathogenic | Endometrial |
| *MSH6* | 8488 | Comprehensive | c.3155_3156delAG | p.Glu1052ValfsX13 | Frameshift | Pathogenic | Colorectal; Skin |
| *MSH6* | 12017 | Comprehensive | c.3155_3156delAG | p.Glu1052ValfsX13 | Frameshift | Pathogenic | Colorectal; Endometrial |
| *MSH6* | 18568 | Breast/Ovarian | c.3155_3156delAG | p.Glu1052ValfsX13 | Frameshift | Pathogenic | Unaffected |
| *MSH6* | 43649 | High/Moderate | c.3155_3156delAG | p.Glu1052ValfsX13 | Frameshift | Pathogenic | Endometrial; Ovarian |
| *MSH6* | 5645 | Lynch/CRC High Risk | c.3202C>T | p.Arg1068Ter | Nonsense | Pathogenic | Endometrial |
| *MSH6* | 21927 | High/Moderate | c.3202C>T | p.Arg1068Ter | Nonsense | Pathogenic | Breast; Endometrial |
| *MSH6* | 32075 | Breast/Ovarian | c.3202C>T | p.Arg1068Ter | Nonsense | Pathogenic | Ovarian |
| *MSH6* | 39139 | Comprehensive | c.3202C>T | p.Arg1068Ter | Nonsense | Pathogenic | Breast; Endometrial |
| *MSH6* | 11577 | Breast/Ovarian | c.3226C>T | p.Arg1076Cys | Missense | Likely Pathogenic | Unaffected |
| *MSH6* | 16480 | Breast/Ovarian | c.3226C>T | p.Arg1076Cys | Missense | Likely Pathogenic | Breast |
| *MSH6* | 17455 | Breast/Ovarian | c.3226C>T | p.Arg1076Cys | Missense | Likely Pathogenic | Ovarian |
| *MSH6* | 24638 | High/Moderate | c.3226C>T | p.Arg1076Cys | Missense | Likely Pathogenic | Unaffected |
| *MSH6* | 43227 | Comprehensive | c.3226C>T | p.Arg1076Cys | Missense | Likely Pathogenic | Unaffected |
| *MSH6* | 162 | Comprehensive | c.3261delC | p.Phe1088SerfsX2 | Frameshift | Pathogenic | Endometrial |
| *MSH6* | 1939 | Comprehensive | c.3261delC | p.Phe1088SerfsX2 | Frameshift | Pathogenic | Breast; Colorectal; Renal |
| *MSH6* | 2747 | Breast/Ovarian | c.3261delC | p.Phe1088SerfsX2 | Frameshift | Pathogenic | Ovarian |
| *MSH6* | 5746 | Breast/Ovarian | c.3261delC | p.Phe1088SerfsX2 | Frameshift | Pathogenic | Ovarian |
| *MSH6* | 8970 | Breast/Ovarian | c.3261delC | p.Phe1088SerfsX2 | Frameshift | Pathogenic | Breast |
| *MSH6* | 22308 | Lynch/CRC High Risk | c.3261delC | p.Phe1088SerfsX2 | Frameshift | Pathogenic | Endometrial |
| *MSH6* | 2093 | Comprehensive | c.3261dupC | p.Phe1088LeufsX5 | Frameshift | Pathogenic | Endometrial |
| *MSH6* | 6701 | Comprehensive | c.3261dupC | p.Phe1088LeufsX5 | Frameshift | Pathogenic | Colorectal |
| *MSH6* | 6861 | High/Moderate | c.3261dupC | p.Phe1088LeufsX5 | Frameshift | Pathogenic | Breast; Renal |
| *MSH6* | 17900 | Breast/Ovarian | c.3261dupC | p.Phe1088LeufsX5 | Frameshift | Pathogenic | Breast |
| *MSH6* | 19589 | Breast/Ovarian | c.3261dupC | p.Phe1088LeufsX5 | Frameshift | Pathogenic | Ovarian |
| *MSH6* | 32838 | Breast/Ovarian | c.3261dupC | p.Phe1088LeufsX5 | Frameshift | Pathogenic | Breast |
| *MSH6* | 46706 | Reflex to Remainder of Comprehensive after Breast Cancer High Risk and PALB2 | c.3261dupC | p.Phe1088LeufsX5 | Frameshift | Pathogenic | Breast |
| *MSH6* | 37978 | Comprehensive | c.3261dupC | p.Phe1088LeufsX5 | Frameshift | Pathogenic | Unaffected |
| *MSH6* | 41202 | Breast/Ovarian | c.3261dupC | p.Phe1088LeufsX5 | Frameshift | Pathogenic | Breast |
| *MSH6* | 24049 | Comprehensive | c.3268_3274delGAGCTTA | p.Glu1090LysfsX23 | Frameshift | Pathogenic | Endometrial |
| *MSH6* | 28694 | Endometrial | c.3379_3438+5del65 | p.Ala1127LeufsX15 | Frameshift | Pathogenic | Endometrial |
| *MSH6* | 8921 | Colorectal | c.3439-2A>G | IVS5-2 A>G | Splice | Pathogenic | Endometrial |
| *MSH6* | 11098 | Comprehensive | c.3439-2A>G | IVS5-2 A>G | Splice | Pathogenic | Endometrial; Sarcoma; Skin (BCC) |
| *MSH6* | 15853 | Endometrial | c.3439-2A>G | IVS5-2 A>G | Splice | Pathogenic | Endometrial |
| *MSH6* | 33076 | High/Moderate | c.3439-2A>G | IVS5-2A>G | Splice | Pathogenic | Sarcoma |
| *MSH6* | 30762 | Comprehensive | c.3513_3514delTA | p.Asp1171GlufsX5 | Frameshift | Pathogenic | Endometrial |
| *MSH6* | 29415 | Endometrial | c.3516_3517delAG | p.Arg1172SerfsX4 | Frameshift | Pathogenic | Unaffected |
| *MSH6* | 3636 | Lynch/CRC High Risk | c.3523_3524dupAC | p.Arg1176LeufsX9 | Frameshift | Pathogenic | Colorectal |
| *MSH6* | 35555 | Colorectal | c.3577_3581delGAATT | p.Glu1193LysfsX2 | Frameshift | Pathogenic | Unaffected |
| *MSH6* | 5242 | Endometrial | c.3690delA | p.Val1231LeufsX9 | Frameshift | Pathogenic | Endometrial |
| *MSH6* | 16645 | Lynch/CRC High Risk | c.3699_3702delAGAA | p.Lys1233AsnfsX6 | Frameshift | Pathogenic | Endometrial |
| *MSH6* | 36948 | Lynch/CRC High Risk | c.3699_3702delAGAA | p.Lys1233AsnfsX6 | Frameshift | Pathogenic | Endometrial |
| *MSH6* | 31127 | Lynch/CRC High Risk | c.3716_3717delTA | p.Ile1239LysfsX35 | Frameshift | Pathogenic | Breast |
| *MSH6* | 16524 | Comprehensive | c.3732_3735dupATTT | p.Ser1246IlefsX30 | Frameshift | Pathogenic | Unaffected |
| *MSH6* | 17088 | Breast/Ovarian | c.3743_3744insT | p.Tyr1249LeufsX26 | Frameshift | Pathogenic | Breast; Endometrial |
| *MSH6* | 1891 | Lynch/CRC High Risk | c.3746_3749dupACCA | p.His1250GlnfsX26 | Frameshift | Pathogenic | Endometrial |
| *MSH6* | 11408 | Endometrial | c.3768T>G | p.Tyr1256Ter | Nonsense | Pathogenic | Endometrial |
| *MSH6* | 40668 | Breast/Ovarian | c.3804dupA | p.Cys1269MetfsX6 | Frameshift | Pathogenic | Breast |
| *MSH6* | 36758 | Lynch/CRC High Risk | c.3840_3846delGGAGACT | p.Glu1281LeufsX44 | Frameshift | Pathogenic | Endometrial |
| *MSH6* | 19827 | Endometrial | c.3882delT | p.Pro1295LeufsX32 | Frameshift | Pathogenic | Endometrial |
| *MSH6* | 21647 | Comprehensive | c.3897_3931dup35 | p.Glu1311AlafsX28 | Frameshift | Pathogenic | Endometrial; Ovarian; Skin (BCC) |
| *MSH6* | 37658 | Lynch Syndrome | c.3932_3935dupAAGT | p.Ile1313SerfsX7 | Frameshift | Pathogenic | Unaffected |
| *MSH6* | 23518 | Endometrial | c.3934_3937dupGTTA | p.Ile1313SerfsX7 | Frameshift | Pathogenic | Endometrial |
| *MSH6* | 17119 | High/Moderate | c.3939_3940dupTC | p.Gln1314LeufsX14 | Frameshift | Pathogenic | Breast |
| *MSH6* | 3955 | Comprehensive | c.3939_3957dup19 | p.Ala1320SerfsX5 | Frameshift | Pathogenic | Endometrial; Ovarian |
| *MSH6* | 14152 | Endometrial | c.3939_3957dup19 | p.Ala1320SerfsX5 | Frameshift | Pathogenic | Endometrial |
| *MSH6* | 32421 | Comprehensive | c.3939_3957dup19 | p.Ala1320SerfsX5 | Frameshift | Pathogenic | Unaffected |
| *MSH6* | 39664 | Comprehensive | c.3939_3957dup19 | p.Ala1320SerfsX5 | Frameshift | Pathogenic | Breast; Colorectal; Endometrial |
| *MSH6* | 41000 | Comprehensive | c.3939_3957dup19 | p.Ala1320SerfsX5 | Frameshift | Pathogenic | Endometrial; Thyroid |
| *MSH6* | 16599 | High/Moderate | c.3959_3962delCAAG | p.Ala1320GlufsX6 | Frameshift | Pathogenic | Breast |
| *MSH6* | 21240 | Breast/Ovarian | c.3959_3962delCAAG | p.Ala1320GlufsX6 | Frameshift | Pathogenic | Breast |
| *MSH6* | 2652 | Lynch/CRC High Risk | c.3984_3987dupGTCA | p.Leu1330AlafsX12 | Frameshift | Pathogenic | Endometrial |
| *MSH6* | 25957 | High/Moderate | c.3984_3987dupGTCA | p.Leu1330ValfsX12 | Frameshift | Pathogenic | Breast |
| *MSH6* | 31017 | Endometrial | c.3984_3987dupGTCA | p.Leu1330ValfsX12 | Frameshift | Pathogenic | Endometrial |
| *MSH6* | 43446 | Comprehensive | c.3984_3987dupGTCA | p.Leu1330ValfsX12 | Frameshift | Pathogenic | Breast; Endometrial |
| *MSH6* | 5844 | Endometrial | c.3991C>T | p.Arg1331Ter | Nonsense | Pathogenic | Breast; Colorectal |
| *MSH6* | 11866 | Colorectal | c.3991C>T | p.Arg1331Ter | Nonsense | Pathogenic | Colorectal |
| *MSH6* | 18228 | Colorectal | c.3991C>T | p.Arg1331Ter | Nonsense | Pathogenic | Colorectal |
| *MSH6* | 21799 | Breast/Ovarian | c.3991C>T | p.Arg1331Ter | Nonsense | Pathogenic | Ovarian |
| *MSH6* | 24876 | Comprehensive | c.3991C>T | p.Arg1331Ter | Nonsense | Pathogenic | Colorectal |
| *MSH6* | 4782 | Endometrial | c.4001G>A | p.Arg1334Gln | Missense | Pathogenic | Breast; Colorectal |
| *MSH6* | 22575 | Colorectal | c.4001G>A | p.Arg1334Gln | Missense | Pathogenic | Unaffected |
| *MSH6* | 11497 | High/Moderate | Deletion Exons 4-7 |  | Large Deletion | Pathogenic | Unaffected |
| *MSH6* | 15449 | Colorectal | Deletion Exons 5-6 |  | Large Deletion | Pathogenic | Endometrial |
| *MSH6* | 26959 | Lynch Syndrome | Deletion Exons 5-6 |  | Large Deletion | Pathogenic | Unaffected |
| *PMS2* | 7123 | Breast/Ovarian | c.1A>G | p.Met1? | Start Codon | Pathogenic | Breast |
| *PMS2* | 14848 | Breast/Ovarian | c.1A>G | p.Met1? | Start Codon | Pathogenic | Breast |
| *PMS2* | 20089 | High/Moderate | c.1A>G | p.Met1? | Start Codon | Pathogenic | Breast |
| *PMS2* | 23137 | Lynch/CRC High Risk | c.1A>G | p.Met1? | Start Codon | Pathogenic | Colorectal |
| *PMS2* | 38611 | Breast/Ovarian | c.1A>G | p.Met1? | Start Codon | Pathogenic | Unaffected |
| *PMS2* | 3494 | Comprehensive | c.2T>A | p.Met1? | Start Codon | Pathogenic | Colorectal |
| *PMS2* | 1060 | Breast/Ovarian | c.2T>C | p.Met1? | Start Codon | Pathogenic | Breast |
| *PMS2* | 30463 | Comprehensive | c.7C>T | p.Arg3Ter | Nonsense | Pathogenic | Unaffected |
| *PMS2* | 338 | Breast/Ovarian | c.137G>T | p.Ser46Ile | Missense | Pathogenic | Unaffected |
| *PMS2* | 4630 | Comprehensive | c.137G>T | p.Ser46Ile | Missense | Pathogenic | Colorectal; Cervical; Melanoma |
| *PMS2* | 5759 | Comprehensive | c.137G>T | p.Ser46Ile | Missense | Pathogenic | Unaffected |
| *PMS2* | 9725 | Colorectal | c.137G>T | p.Ser46Ile | Missense | Pathogenic | Colorectal |
| *PMS2* | 13988 | Breast/Ovarian | c.137G>T | p.Ser46Ile | Missense | Pathogenic | Breast |
| *PMS2* | 15673 | Lynch/CRC High Risk | c.137G>T | p.Ser46Ile | Missense | Pathogenic | Colorectal |
| *PMS2* | 18133 | Comprehensive | c.137G>T | p.Ser46Ile | Missense | Pathogenic | Unaffected |
| *PMS2* | 18817 | High/Moderate | c.137G>T | p.Ser46Ile | Missense | Pathogenic | Breast |
| *PMS2* | 25550 | Lynch Syndrome | c.137G>T | p.Ser46Ile | Missense | Pathogenic | Unaffected |
| *PMS2* | 26810 | Comprehensive | c.137G>T | p.Ser46Ile | Missense | Pathogenic | Unaffected |
| *PMS2* | 29731 | Lynch/CRC High Risk | c.137G>T | p.Ser46Ile | Missense | Pathogenic | Unaffected |
| *PMS2* | 33311 | Breast/Ovarian | c.137G>T | p.Ser46Ile | Missense | Pathogenic | Breast |
| *PMS2* | 34505 | High/Moderate | c.137G>T | p.Ser46Ile | Missense | Pathogenic | Breast |
| *PMS2* | 37548 | Lynch/CRC High Risk | c.137G>T | p.Ser46Ile | Missense | Pathogenic | Unaffected |
| *PMS2* | 37761 | Breast/Ovarian | c.137G>T | p.Ser46Ile | Missense | Pathogenic | Unaffected |
| *PMS2* | 42599 | Lynch/CRC High Risk | c.137G>T | p.Ser46Ile | Missense | Pathogenic | Colorectal |
| *PMS2* | 43103 | Comprehensive | c.137G>T | p.Ser46Ile | Missense | Pathogenic | Colorectal |
| *PMS2* | 43128 | High/Moderate | c.137G>T | p.Ser46Ile | Missense | Pathogenic | Unaffected |
| *PMS2* | 43355 | Breast/Ovarian | c.137G>T | p.Ser46Ile | Missense | Pathogenic | Breast |
| *PMS2* | 43470 | Lynch Syndrome | c.137G>T | p.Ser46Ile | Missense | Pathogenic | Colorectal |
| *PMS2* | 32820 | High/Moderate | c.139_146delCTGGATGC | p.Leu47TrpfsX4 | Frameshift | Likely Pathogenic | Breast |
| *PMS2* | 26465 | High/Moderate | c.142delG | p.Asp48MetfsX9 | Frameshift | Pathogenic | Colorectal |
| *PMS2* | 40957 | Breast/Ovarian | c.23+1G>T | IVS1+1 G>T | Splice | Pathogenic | Breast |
| *PMS2* | 43018 | Comprehensive | c.247_250dupTTAA | p.Thr84IlefsX9 | Frameshift | Pathogenic | Breast; Endometrial |
| *PMS2* | 24519 | High/Moderate | c.325dupG | p.Glu109GlyfsX30 | Frameshift | Pathogenic | Unaffected |
| *PMS2* | 46705 | Reflex to  Remainder of Comprehensive  after Breast Cancer High Risk | c.445delT | p.Tyr149ThrfsX52 | Frameshift | Pathogenic | Breast |
| *PMS2* | 22761 | High/Moderate | c.631C>T | p.Arg211Ter | Nonsense | Pathogenic | Breast; Carcinoid Tumor |
| *PMS2* | 27038 | Comprehensive | c.631C>T | p.Arg211Ter | Nonsense | Pathogenic | Endometrial |
| *PMS2* | 905 | Breast/Ovarian | c.697C>T | p.Gln233Ter | Nonsense | Pathogenic | Unaffected |
| *PMS2* | 11632 | Breast/Ovarian | c.736_741delCCCCCTins11 | p.Pro246CysfsX3 | Frameshift | Pathogenic | Unaffected |
| *PMS2* | 17294 | Comprehensive | c.736_741delCCCCCTins11 | p.Pro246CysfsX3 | Frameshift | Pathogenic | Unaffected |
| *PMS2* | 17395 | Breast/Ovarian | c.736_741delCCCCCTins11 | p.Pro246CysfsX3 | Frameshift | Pathogenic | Unaffected |
| *PMS2* | 22285 | High/Moderate | c.736_741delCCCCCTins11 | p.Pro246CysfsX3 | Frameshift | Pathogenic | Endometrial |
| *PMS2* | 22900 | Breast/Ovarian | c.736_741delCCCCCTins11 | p.Pro246CysfsX3 | Frameshift | Pathogenic | Unaffected |
| *PMS2* | 26249 | Breast/Ovarian | c.736_741delCCCCCTins11 | p.Pro246CysfsX3 | Frameshift | Pathogenic | Unaffected |
| *PMS2* | 27956 | Breast/Ovarian | c.736_741delCCCCCTins11 | p.Pro246CysfsX3 | Frameshift | Pathogenic | Unaffected |
| *PMS2* | 28309 | Breast/Ovarian | c.736_741delCCCCCTins11 | p.Pro246CysfsX3 | Frameshift | Pathogenic | Breast |
| *PMS2* | 31885 | Breast/Ovarian | c.736_741delCCCCCTins11 | p.Pro246CysfsX3 | Frameshift | Pathogenic | Breast |
| *PMS2* | 32822 | Breast/Ovarian | c.736_741delCCCCCTins11 | p.Pro246CysfsX3 | Frameshift | Pathogenic | Breast |
| *PMS2* | 46704 | Reflex to  Remainder of Comprehensive  after Breast Cancer High Risk | c.736_741delCCCCCTinsTGTGTGTGAAG | p.Pro246CysfsX3 | Frameshift | Pathogenic | Breast |
| *PMS2* | 7780 | Comprehensive | c.736_741delCCCCCTinsTGTGTGTGAAG | p.Pro246CysfsX3 | Frameshift | Pathogenic | Colorectal; Ovarian |
| *PMS2* | 13387 | Lynch/CRC High Risk | c.765C>A | p.Tyr255Ter | Nonsense | Pathogenic | Colorectal; Endometrial |
| *PMS2* | 25896 | Comprehensive | c.765C>A | p.Tyr255Ter | Nonsense | Pathogenic | Endometrial |
| *PMS2* | 29010 | Comprehensive | c.809C>G | p.Ser270Ter | Nonsense | Pathogenic | Unaffected |
| *PMS2* | 1204 | Endometrial | c.823C>T | p.Gln275Ter | Nonsense | Pathogenic | Endometrial |
| *PMS2* | 30654 | Endometrial | c.823C>T | p.Gln275Ter | Nonsense | Pathogenic | Endometrial |
| *PMS2* | 34965 | Breast/Ovarian | c.851delC | p.Ser284Ter | Nonsense | Pathogenic | Unaffected |
| *PMS2* | 8587 | Endometrial | c.861_864delACAG | p.Arg287SerfsX19 | Frameshift | Pathogenic | Endometrial |
| *PMS2* | 12064 | Breast/Ovarian | c.861_864delACAG | p.Arg287SerfsX19 | Frameshift | Pathogenic | Unaffected |
| *PMS2* | 23057 | Comprehensive | c.861_864delACAG | p.Arg287SerfsX19 | Frameshift | Pathogenic | Skin (BCC) |
| *PMS2* | 38010 | Comprehensive | c.903G>T | p.Lys301Asn | Cryptic Splice | Likely Pathogenic | Colorectal; Endometrial |
| *PMS2* | 1760 | Comprehensive | c.943C>T | p.Arg315Ter | Nonsense | Pathogenic | Unaffected |
| *PMS2* | 3916 | Breast/Ovarian | c.943C>T | p.Arg315Ter | Nonsense | Pathogenic | Breast |
| *PMS2* | 1382 | Lynch/CRC High Risk | c.989-1G>T | IVS9-1 G>T | Splice | Pathogenic | Unaffected |
| *PMS2* | 43480 | Colorectal | c.989-1G>T | IVS9-1 G>T | Splice | Pathogenic | Colorectal |
| *PMS2* | 33431 | Breast/Ovarian | c.1021delA | p.Arg341GlyfsX15 | Frameshift | Pathogenic | Unaffected |
| *PMS2* | 17021 | High/Moderate | c.1067delA | p.Lys356ArgfsX4 | Frameshift | Pathogenic | Endometrial |
| *PMS2* | 2496 | Breast/Ovarian | c.1112_1113delATinsTTTA | p.Asn371IlefsX2 | Frameshift | Pathogenic | Breast |
| *PMS2* | 36427 | High/Moderate | c.1144+1G>A | IVS10+1 G>A | Splice | Pathogenic | Colorectal |
| *PMS2* | 2999 | Lynch/CRC High Risk | c.1239delA | p.Asp414ThrfsX34 | Frameshift | Pathogenic | Endometrial |
| *PMS2* | 32589 | Comprehensive | c.1239dupA | p.Asp414ArgfsX44 | Frameshift | Pathogenic | Breast; Endometrial |
| *PMS2* | 21803 | Comprehensive | c.1261C>T | p.Arg421Ter | Nonsense | Pathogenic | Breast |
| *PMS2* | 31918 | Breast/Ovarian | c.1261C>T | p.Arg421Ter | Nonsense | Pathogenic | Breast |
| *PMS2* | 35246 | Breast/Ovarian | c.1261C>T | p.Arg421Ter | Nonsense | Pathogenic | Breast |
| *PMS2* | 34636 | Comprehensive | c.1576delG | p.Asp526ThrfsX69 | Frameshift | Pathogenic | Breast |
| *PMS2* | 36604 | High/Moderate | c.1576delG | p.Asp526ThrfsX69 | Frameshift | Pathogenic | Unaffected |
| *PMS2* | 3627 | Comprehensive | c.1579_1580delAG | p.Arg527GlyfsX14 | Frameshift | Pathogenic | Breast |
| *PMS2* | 9839 | Comprehensive | c.1687C>T | p.Arg563Ter | Nonsense | Pathogenic | Breast |
| *PMS2* | 13407 | Colorectal | c.1687C>T | p.Arg563Ter | Nonsense | Pathogenic | Colorectal |
| *PMS2* | 24497 | Breast/Ovarian | c.1687C>T | p.Arg563Ter | Nonsense | Pathogenic | Breast |
| *PMS2* | 29018 | Comprehensive | c.1687C>T | p.Arg563Ter | Nonsense | Pathogenic | Unaffected |
| *PMS2* | 8591 | Breast/Ovarian | c.1831dupA | p.Ile611AsnfsX2 | Frameshift | Pathogenic | Fallopian Tube; Skin (SCC) |
| *PMS2* | 21049 | Breast/Ovarian | c.1831dupA | p.Ile611AsnfsX2 | Frameshift | Pathogenic | Unaffected |
| *PMS2* | 42632 | Comprehensive | c.1831dupA | p.Ile611AsnfsX2 | Frameshift | Pathogenic | Unaffected |
| *PMS2* | 11217 | Breast/Ovarian | c.1840A>T | p.Lys614Ter | Nonsense | Pathogenic | Breast |
| *PMS2* | 31495 | Comprehensive | c.1840A>T | p.Lys614Ter | Nonsense | Pathogenic | Breast |
| *PMS2* | 13004 | Breast/Ovarian | c.1874delT | p.Leu625Ter | Nonsense | Pathogenic | Unaffected |
| *PMS2* | 14890 | High/Moderate | c.1882C>T | p.Arg628Ter | Nonsense | Pathogenic | Breast |
| *PMS2* | 23189 | Comprehensive | c.1882C>T | p.Arg628Ter | Nonsense | Pathogenic | Breast |
| *PMS2* | 33888 | Comprehensive | c.1927C>T | p.Gln643Ter | Nonsense | Pathogenic | Unaffected |
| *PMS2* | 34615 | High/Moderate | c.1927C>T | p.Gln643Ter | Nonsense | Pathogenic | Colorectal |
| *PMS2* | 37994 | High/Moderate | c.1970dupA | p.Asn657LysfsX7 | Frameshift | Pathogenic | Endometrial |
| *PMS2* | 9935 | Lynch/CRC High Risk | c.2095G>C | p.Asp699His | Missense | Likely Pathogenic | Colorectal |
| *PMS2* | 30891 | Breast/Ovarian | c.2095G>C | p.Asp699His | Missense | Likely Pathogenic | Unaffected |
| *PMS2* | 5017 | Breast/Ovarian | c.2113G>A | p.Glu705Lys | Missense | Pathogenic | Breast |
| *PMS2* | 6934 | Comprehensive | c.2117delA | p.Lys706SerfsX19 | Frameshift | Pathogenic | Ovarian |
| *PMS2* | 10741 | Comprehensive | c.2117delA | p.Lys706SerfsX19 | Frameshift | Pathogenic | Breast |
| *PMS2* | 22174 | Breast/Ovarian | c.2117delA | p.Lys706SerfsX19 | Frameshift | Pathogenic | Breast |
| *PMS2* | 14523 | Endometrial | c.2156delA | p.Gln719ArgfsX6 | Frameshift | Pathogenic | Endometrial |
| *PMS2* | 42003 | Breast/Ovarian | c.2156delA | p.Gln719ArgfsX6 | Frameshift | Pathogenic | Breast |
| *PMS2* | 21976 | High/Moderate | c.2192_2196delTAACT | p.Leu731CysfsX3 | Frameshift | Pathogenic | Unaffected |
| *PMS2* | 8797 | Breast/Ovarian | c.2404C>T | p.Arg802Ter | Nonsense | Pathogenic | Unaffected |
| *PMS2* | 15907 | Lynch/CRC High Risk | c.2404C>T | p.Arg802Ter | Nonsense | Pathogenic | Colorectal |
| *PMS2* | 20011 | Breast/Ovarian | c.2404C>T | p.Arg802Ter | Nonsense | Pathogenic | Unaffected |
| *PMS2* | 41622 | Colorectal | c.2404C>T | p.Arg802Ter | Nonsense | Pathogenic | Colorectal |
| *PMS2* | 18216 | High/Moderate | c.2500_2501delATinsG | p.Met834GlyfsX17 | Frameshift | Likely Pathogenic | Ovarian |
| *PMS2* | 30518 | Breast/Ovarian | c.2500_2501delATinsG | p.Met834GlyfsX17 | Frameshift | Likely Pathogenic | Unaffected |
| *PMS2* | 32623 | Comprehensive | c.2522G>A | p.Trp841Ter | Nonsense | Likely Pathogenic | Unaffected |
| *PMS2* | 11074 | Comprehensive | Deletion Exons 1-10 |  | Large Deletion | Pathogenic | Breast; Endometrial |
| *PMS2* | 18678 | Breast/Ovarian | Deletion Exons 5-7 |  | Large Deletion | Pathogenic | Breast; Endometrial |
| *PMS2* | 3909 | Comprehensive | Deletion Exons 5-9 |  | Large Deletion | Pathogenic | Breast; Colorectal |
| *PMS2* | 14281 | Breast/Ovarian | Deletion Exons 5-9 |  | Large Deletion | Pathogenic | Endometrial |
| *PMS2* | 33830 | Breast/Ovarian | Deletion Exons 6-7 |  | Large Deletion | Pathogenic | Breast |
| *PMS2* | 34697 | Comprehensive | Deletion Exons 6-7 |  | Large Deletion | Pathogenic | Unaffected |
| *PMS2* | 2386 | Comprehensive | Deletion Exons 6-8 |  | Large Deletion | Pathogenic | Colorectal; Cervical |
| *PMS2* | 42416 | Breast/Ovarian | Deletion Exons 6-8 |  | Large Deletion | Pathogenic | Breast |
| *PMS2* | 29944 | Lynch Syndrome | Deletion Exons 6-9 |  | Large Deletion | Pathogenic | Unaffected |
| *PMS2* | 8563 | Comprehensive | Deletion Exons 7-8 |  | Large Deletion | Pathogenic | Unaffected |
| *PMS2* | 1377 | Comprehensive | Deletion Exon 8 |  | Large Deletion | Pathogenic | Endometrial |
| *PMS2* | 12067 | Comprehensive | Deletion Exon 8 |  | Large Deletion | Pathogenic | Unaffected |
| *PMS2* | 15898 | Comprehensive | Deletion Exon 8 |  | Large Deletion | Pathogenic | Breast; Endometrial |
| *PMS2* | 20718 | Breast/Ovarian | Deletion Exon 8 |  | Large Deletion | Pathogenic | Breast |
| *PMS2* | 34114 | High/Moderate | Deletion Exons 8-10 |  | Large Deletion | Pathogenic | Endometrial |
| *PMS2* | 9668 | Comprehensive | Deletion Exons 9-10 |  | Large Deletion | Pathogenic | Unaffected |
| *PMS2* | 3413 | Colorectal | Deletion Exon 10 |  | Large Deletion | Pathogenic | Unaffected |
| *PMS2* | 14541 | Breast/Ovarian | Deletion Exon 10 |  | Large Deletion | Pathogenic | Brain; Breast |
| *CRC: colorectal cancer; SCC: squamous cell carcinoma; BCC: basal cell carcinoma* | | | | | | | |
