# Supplementary figures and images for: MSH6 and PMS2 germ-line pathogenic variants implicated in Lynch syndrome are associated with breast cancer
Source: Genet Med. 2018 Jan 18;20(10):1167–74. doi: 10.1038/gim.2017.254 (PMC6051923; doi:10.1038/gim.2017.254)

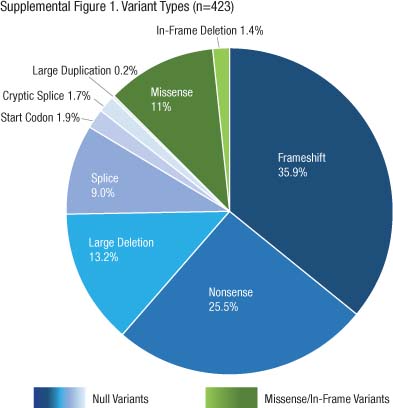

Supplement: Supplementary file 2 — Supplementary Figure 1 [file 41436_2018_201_MOESM2_ESM.jpg]
